# Experimental Study on the Dry Drilling Nickel-Based Superalloy of CrAlYN Coated Carbide Bit

**DOI:** 10.3390/ma15124302

**Published:** 2022-06-17

**Authors:** Hui Li, Feng Gao, Yan Li, Lijing Bai

**Affiliations:** 1 Key Laboratory of NC Machine Tools and Integrated Manufacturing Equipment of the Ministry of Education, Xi’an University of Technology, Xi’an 710048, China; lihui20221982@163.com (H.L.); liyangf@xaut.edu.cn (Y.L.); 2 Department of Mechanical, Henan University of Engineering, Zhengzhou 451191, China; 3 School of Materials Science and Engineering, Xi’an University of Technology, Xi’an 710048, China; bljlxm@xaut.edu.cn

**Keywords:** nickel-based superalloy, dry drilling, CrAlYN coated, carbide, bit service life

## Abstract

Nickel-based superalloy is regarded as one of the materials with the poorest cutting and drilling performance. Additionally, there is much less research on the drilling of it. This paper aims to study the drilling performance of dry drilling nickel-based superalloy with uncoated and CrAlYN coated carbide bit. First of all, the primary and secondary factors influencing the machining performance of dry drilling nickel-base superalloy uncoated carbide bit were explored through an orthogonal test. Secondly, the self-prepared CrAlYN coated carbide drills, and uncoated drills were compared and analyzed from perspectives of service life, drilling force, drilling temperature, drill surface topography, failure mechanism, and machining surface quality. The research results are as follows: the drilling temperature is the primary factor affecting the drilling performance under dry drilling conditions. CrAlYN coating can obviously prolong the service life of tools, reduce the drilling force and drilling temperature, and improve the machining surface quality at lower rotational speeds. Moreover, the coated cemented carbide bit has a similar failure mode to the uncoated cemented carbide bit after the CrAlYN coating falls off in the wear zone of cemented carbide bit, which is mainly bonding wear on the rear tool surface and the front tool surface.

## 1. Introduction

Nickel-based superalloy has been extensively applied in aerospace, nuclear energy, and petroleum industry [1,2,3,4,5] due to relatively strong corrosion resistance and high-temperature oxidation resistance. The refractory metals (molybdenum, niobium, tungsten, etc.) in the nickel-based superalloy are featured by hard spots, high strength, and poor heat dissipation. Thus, the nickel-based superalloy has poorer cutting and drilling performance, which is mainly manifested by difficult chip breaking during machining, serious cutting-tool wear, and too fast temperature rising during cutting and drilling [6,7,8]. Drilling is one of the most important working procedures and is often the last one in aerospace manufacturing. Compared with other cutting and drilling processes [9,10,11,12,13,14,15,16,17], there is less literature about nickel-based superalloy drilling. A nickel-based superalloy is featured by high strength, poor heat dissipation, and many hard spots, which result in short service life and poor processing quality of cutting tools in the process of mechanical drilling. As components miniaturize and the material is applied in a larger range, drilling becomes increasingly important in a variety of applications. At present, a larger number of processing methods are based on hot material removal mechanisms, such as EDM [18,19,20] and laser drilling [21,22,23], but fewer ones are based on mechanical drilling, mainly because of its cutting tool having a shorter service life.

Sharman Arc [24] designed a series of experiments in detail to examine the tool life/wear of commercially available drills for nickel-based superalloys, and he concluded that currently, commercially available drills fail to produce top-class holes to meet the requirements of the aerospace industry. Eckstein M et al. [25] processed holes with a diameter of 8.5 mm through an internally cooled cemented carbide twist drill and found that the tool wear reached the failure standard after processing 24 holes. Kannan S [26] processed holes with a diameter of 12.99 mm by using an internally cooled cemented carbide twist drill and found that the tool surface wear affected the processing quality after processing 40 holes. Based on the related experiments by drilling nickel-based superalloy with high cobalt HSS drill and carbide drill, respectively, Sun Jinliang [27] conducted that the high cobalt HSS drill lost its machining capacity when the machining depth was about 5–6 mm, and carbide bit could process 20 holes at most when the machining depth was 5 mm. Obviously, nickel-based superalloy has poor drilling performance, and the cutting tool made of it has a short service life. Therefore, it is of great significance to study its drilling performance.

In order to reduce production costs and guarantee environmental safety during processing, in recent years, it has become a trend to reduce the use of drilling fluid and turn to dry drilling. The application of coating technology can also improve the quality of machining surfaces [28,29]. R Ramanujam [30] modeled and optimized drilling parameters in dry turning of nickel-based superalloy with coated carbide blades. Umbrello [31] investigated the surface integrity in dry machining of a nickel-base superalloy. D G. Lecoz [32] studied the temperature variation in the workpiece and in the cutting tool during dry milling of nickel-based superalloy and concluded that cutting temperature was a vital parameter in controlling tool service life and machined surface quality. S Yan [33] explored the coating tool temperature variation by establishing a thermal model of coating tool temperature variation in the dry milling of nickel-base superalloy on turbine blades.

In this paper, first of all, the orthogonal test of dry drilling of nickel-based superalloy with cemented carbide bit was conducted to explore the primary and secondary factors influencing the tool service life under dry drilling conditions. Secondly, the dry drilling between self-made CrAlYN coating [34,35] and uncoated cemented carbide drill was compared under selected working conditions. Moreover, the drilling performance of CrAlYN coated carbide bit in dry drilling nickel-base superalloy was discussed.

## 2. Experiment Equipment and Method

### 2.1. Experiment Materials

(1) Nickel-base superalloy

The nickel-base superalloy GH4169 plate was selected as the workpiece material, with a thickness of 18 mm and a length and width of 200 mm. Its chemical composition is shown in Table 1, and the material properties of the workpiece are shown in Table 2.

(2) Drill

**Figure 1:**
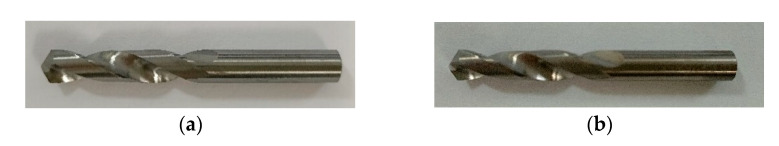
Bits. (**a**) Uncoated bit. (**b**) CrAlYN coated bit.

The brand YG10 cemented carbide in Chinese was selected because of its quite excellent wear resistance, toughness, and strength. As shown in Figure 1, an uncoated YG10 cemented carbide drill with a diameter of 6 mm and a CrAlYN coated drill were selected as the sample base material in this experiment. 

The YG10 cemented carbide is a tungsten cobalt material, in which the Co content accounted for 10%. Table 3 shows the chemical composition of carbide drill YG10, and Table 4 shows its mechanical properties.

The cemented carbide bits YG10 were coated with CrAlYN coated by MSIP016 closed field non-equilibrium magnetron sputtering ion plating equipment of the Xi’an University of Technology. The CrAlYN with a Y content of 3.28% was selected in this experiment, with the elemental mass fraction shown in Table 5. The CrAlYN coated is uniform and dense particle distribution and flat and smooth coating surface [34,35].

### 2.2. Experiment Method

Firstly, uncoated carbide bit YG10 was selected to drill nickel-base superalloy GH4169. By considering the impact of spindle rotating speed, feed rate, and drilling depth on drilling performance, the three-factor and three-level orthogonal test was designed, with the design scheme shown in Table 6. In order to avoid the randomness of experimental results, each group of experiments was repeated three times, and the average was taken. Then, the better working conditions of the uncoated YG10 drill bit were selected, and the self-made CrAlYN coated YG10 drill bit was used for comparative analysis in the drilling experiment to study the drilling performance and friction wear mechanism of nickel-based superalloy.

### 2.3. Experiment Equipment

**Figure 2:**
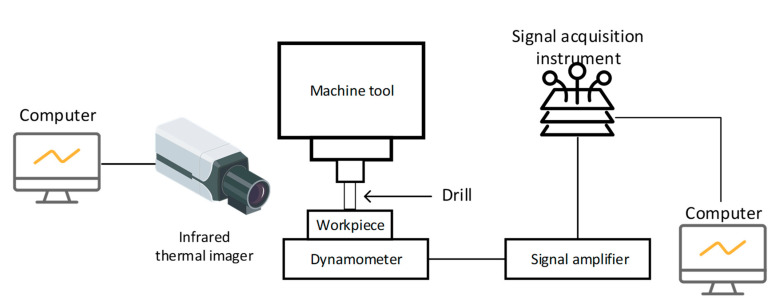
Schematic diagram of drilling experiment.

The experiment was conducted on the SINUMERIK840D CNC programmed five-axis milling machine. A maximum speed of 2000 rpm was set to the machine tool. In the process of drilling, the drilling force was measured through the kistler-9271A force sensor, and the processing temperature was measured by the A315 online infrared thermal imaging instrument. Moreover, the real-time temperature change curve was output by connecting with the computer. The principle of the drilling experiment is shown in Figure 2.

The wear quantity VB at the edge of the back tool surface of more than 0.3 mm was regarded as the standard of being blunt. The wear of the bit was measured through a tool microscope 30 times. The wear of each two holes was measured until the blunt standard was reached. After the drilling experiment, the bit morphology was observed by THE Quanta 250 SEM produced by Czech FEI Company, and the element composition was analyzed by the matched energy spectrum analyzer. The surface roughness Ra was measured five times by the TR200 surface roughness meter, and the average value was calculated.

## 3. Experiment Results and Analysis

### 3.1. Dry Drilling Experiment of Uncoated Carbide Bit

**Figure 3:**
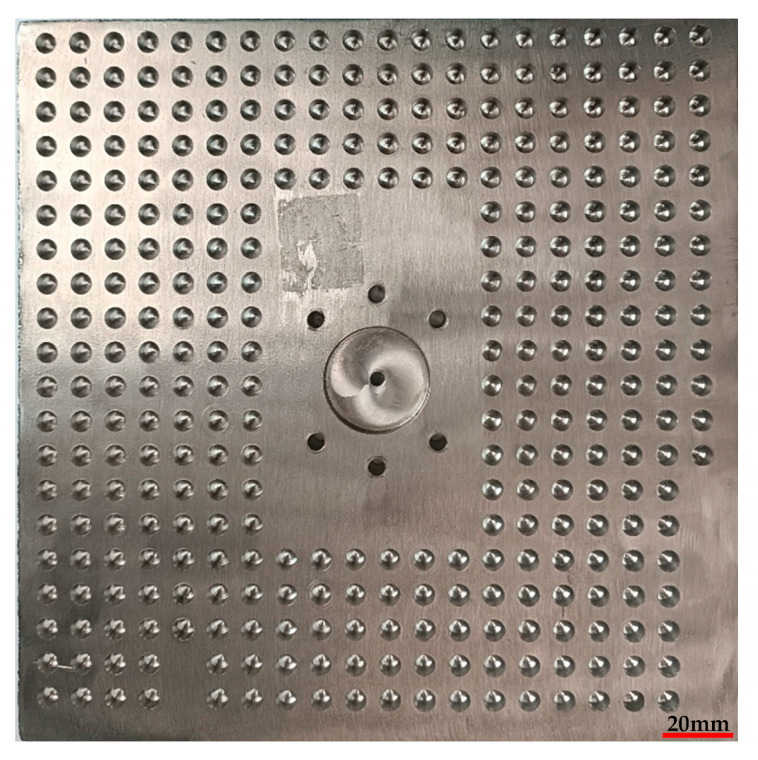
Drilling experiment workpiece.

The three-factor and three-level orthogonal drilling experiment was conducted by using uncoated cemented carbide bit YG10. Every experiment was performed three times and averaged. The drilling experiment workpiece is shown in Figure 3, and the experiment results are shown in Table 7. The number of holes under different working conditions represented the bit service life. Additionally, the average value of the steady state of the axial cutting force and the maximum temperature during drilling represented the drilling force and drilling temperature, respectively. 

The range analysis of bit service life, drilling force, and drilling temperature was conducted, respectively, as shown in Table 8, Table 9 and Table 10. Obviously, the primary factor influencing bit service life and drilling temperature was drilling depth, followed by feed rate and spindle speed. Moreover, the primary factor influencing drilling force were drilling speed, drilling depth, and feed rate.

Under normal circumstances, drilling is a semi-closed chip. The heat entering into the bit accounts for about 52.5%; the heat taken away by chips accounts for 28%; the heat transferring to the workpiece accounts for 14.5%; the other 5% of heat transfers to the surrounding medium. Nickel-based superalloy contains hard points, has high strength, and has poor heat dissipation, which results in large drilling heat and slow heat dissipation. As the hole’s depth becomes larger, there is more heat accumulation and over-high temperature, making the hard alloy drill tend to be damaged and affecting the service life of the bit. Therefore, the drilling temperature is a key factor influencing the tool service life of the nickel-base superalloy drill.

For a too-low drilling feed rate, there would be a longer drilling time for the same drilling depth and a higher drilling temperature. For a too-high drilling feed rate, there would be a short drilling time but larger cutting force, friction and heat, and higher drilling temperature. When the feed rate was 0.04 mm/r, the bit had longer service life and lower drilling temperature. The higher the drilling speed was, the greater the drilling force was and the greater the friction force was. When the drilling speed was 15 m/min, the bit had longer service life and lower drilling temperature. 

Therefore, according to the bit service life and drilling temperature, and combined with the actual drilling depth, the comparison drilling experiments of CrAlYN coated carbide bit and uncoated carbide bit were conducted under two working conditions, namely, drilling depth of 6 mm, drilling feed rate of 0.04 mm/r, and the drilling speed of 15 m/min and 20 m/min, respectively. 

### 3.2. Dry Drilling Experiment of CrAlYN Coated Carbide Bit

**Figure 4:**
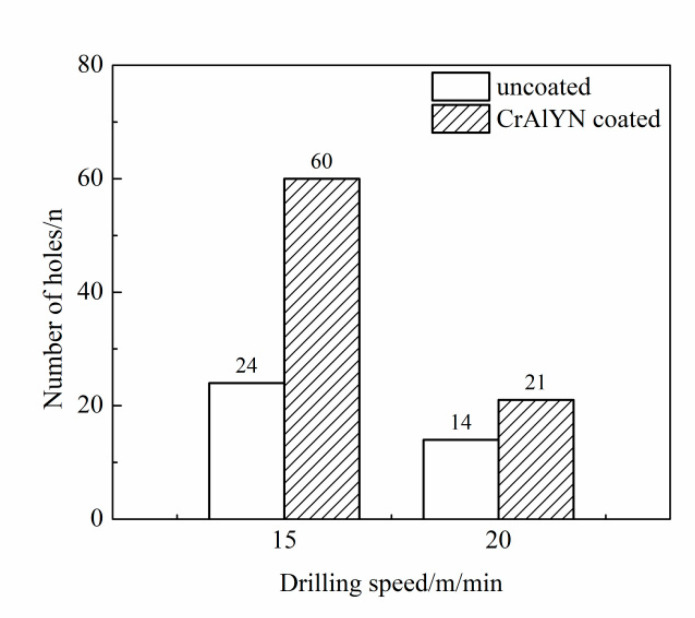
Service life of coated and uncoated drills (drilling depth of 6 mm and drilling feed rate of 0.04 mm/r).

The comparison drilling experiments of CrAlYN coated carbide bit and uncoated carbide bit was conducted under the drilling speed of 15 m/min and 20 m/min, respectively. The experiment results are shown in Figure 4. At the drilling speed of 15 m/min, the uncoated bit and the coated bit drilled 24 holes and 60 holes, respectively, increasing the tool service life by 150%. At the drilling speed of 20 m/min, the uncoated bit and the coated bit drilled 14 holes and 21 holes, respectively, increasing the tool service life by 50%. Therefore, CrAlYN coating could improve the drilling performance and tool service life, especially at lower speeds.

(1) Comparison of drilling force

**Figure 5:**
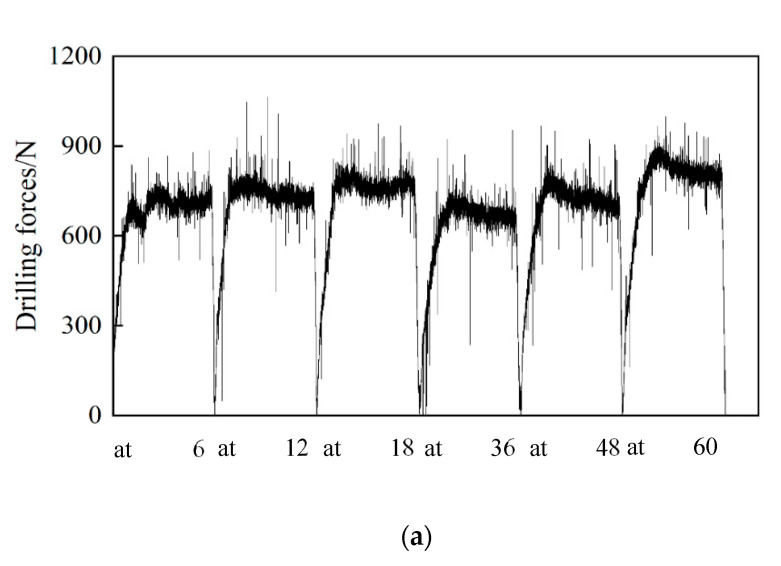

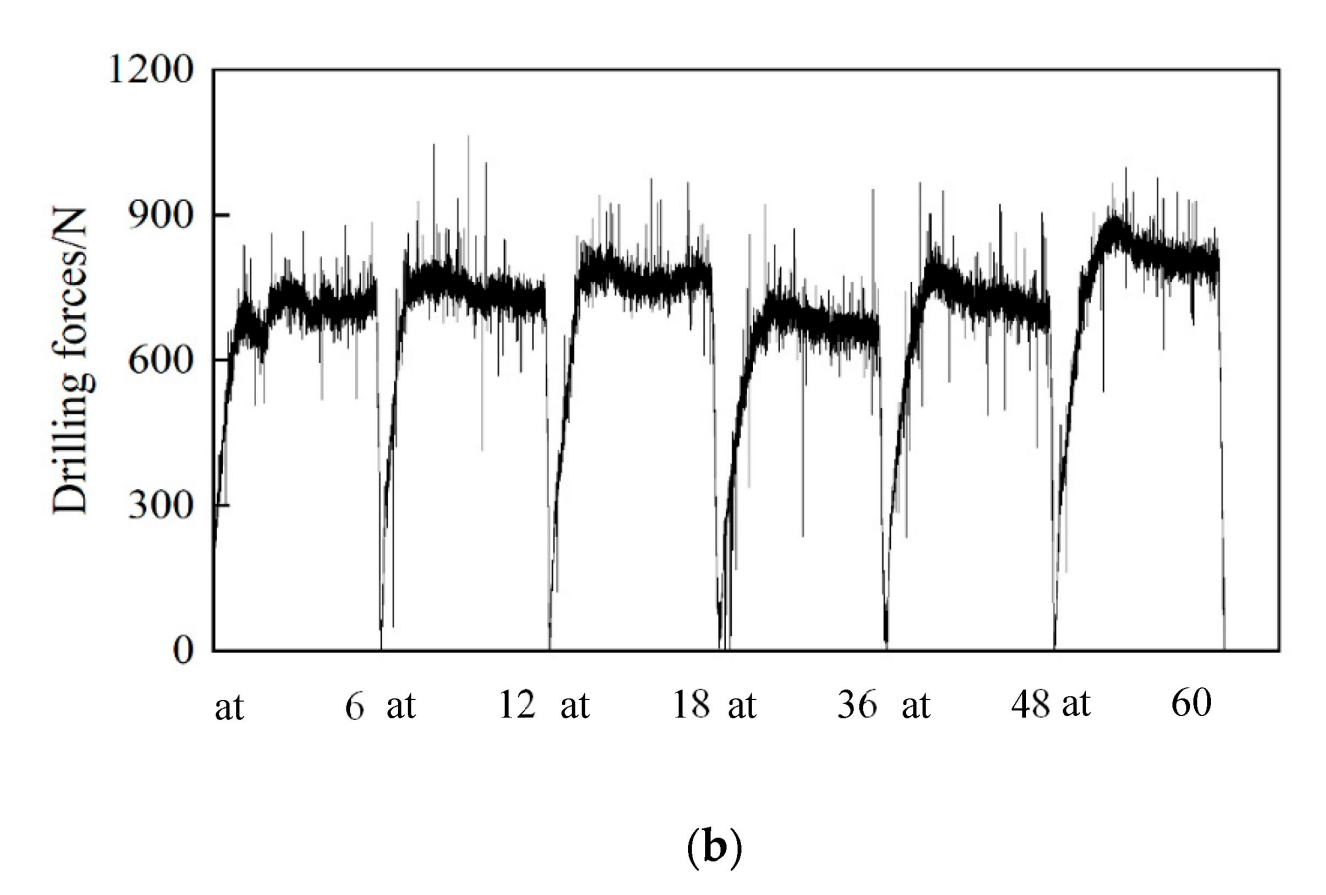
Comparison of drilling forces at drilling speed of 15 m/min, feed rate of 0.04 mm/r, and drilling depth of 6 mm. (**a**) Drilling forces of uncoated bit. (**b**) Drilling forces of coated bit.

Figure 5 shows the comparison of drilling forces of CrAlYN coated and uncoated cemented carbide bit drilling nickel-base superalloy GH4169 at a drilling speed of 15 m/min, feed rate of 0.04 mm/r, and drilling depth of 6 mm. As shown in Figure 5a, the drilling force of the CrAlYN coated bit is approximately 40% less than that of the uncoated bit. The CrAlYN coating makes the bit surface smoother, resulting in less friction.

As seen in Figure 5, although the overall drilling force increased with the number of holes, it was not linear. Sometimes it would decrease a little with the increase in the number of holes, and then the drilling force would significantly increase after drilling a few more holes because the bit cutting edge slowly wear would make the drilling force slowly increase, but the sudden tipping of the edge would make the drilling force rise sharply; with the continued drilling, the tipping of the edge was slowly ground smooth, reducing the roughness, and making the drilling force slightly decrease.

**Figure 6:**
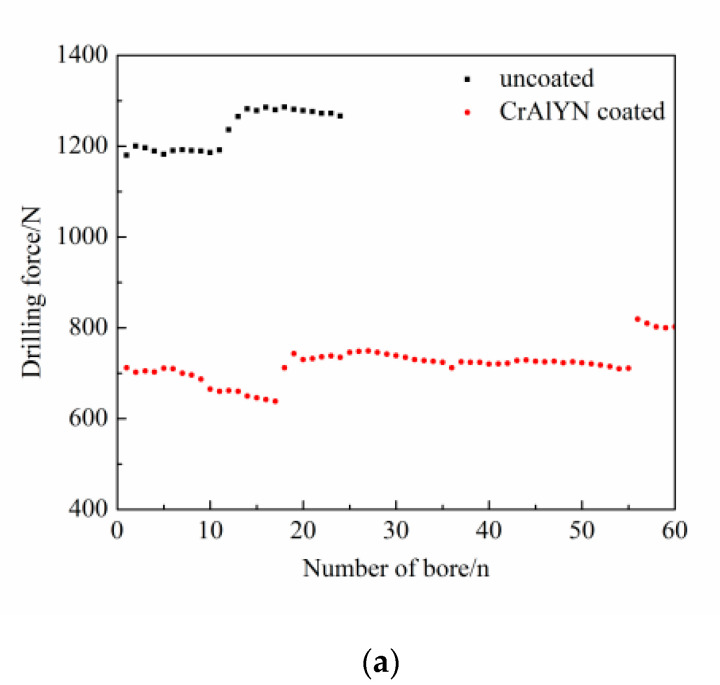

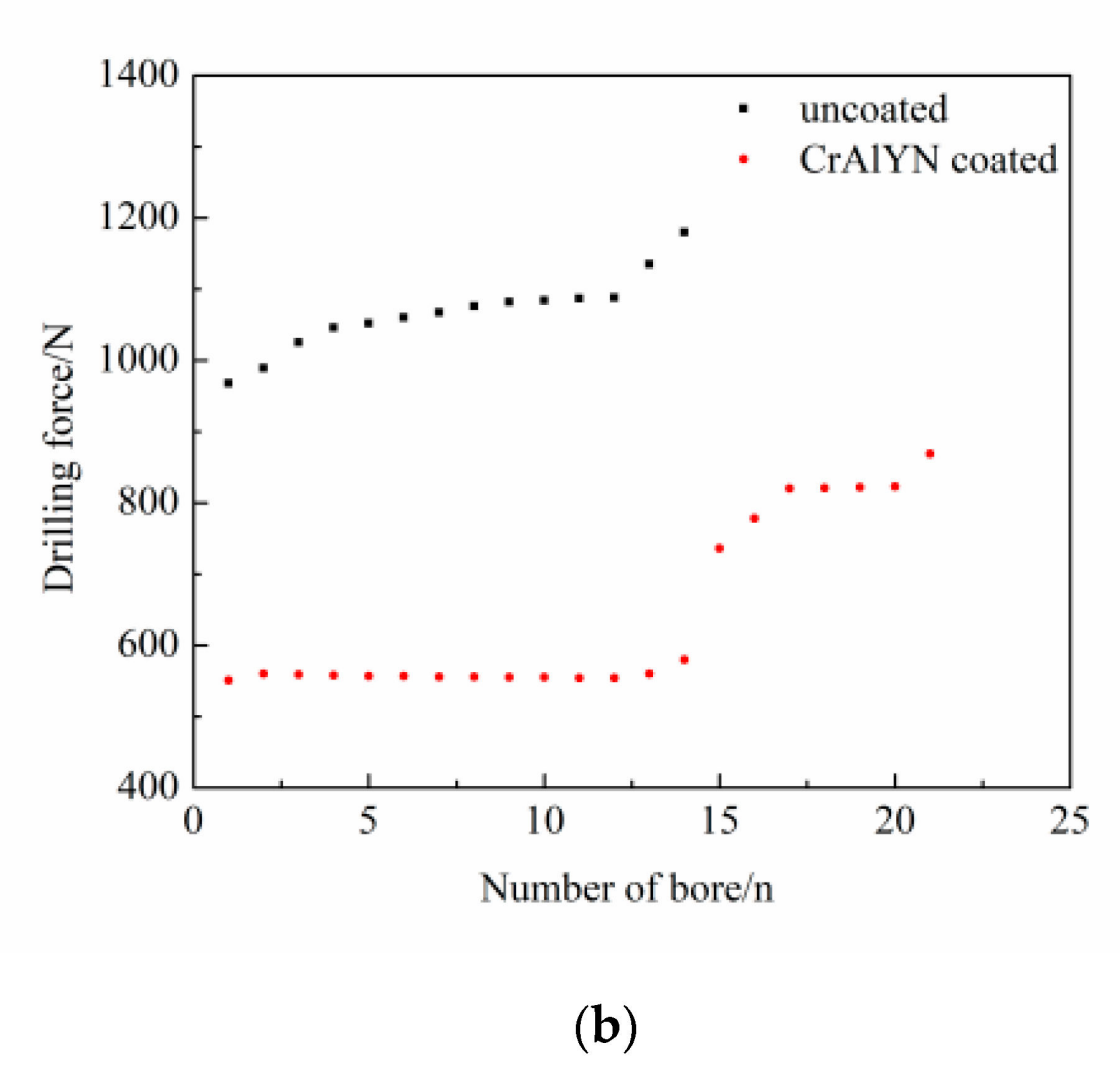
Comparison of drilling force between uncoated and coated bits (drilling depth of 6 mm and drilling feed rate of 0.04 mm/r). (**a**) Drilling force at a drilling speed of 15 m/min. (**b**) Drilling force at a drilling speed of 20 m/min.

Figure 6 shows the comparison curves of drilling force between CrAlYN coated and uncoated cemented carbide bits under two working conditions. As it shows, compared with the uncoated bit, the drilling force of the CrAlYN coated carbide bit decreased by about 40%. The CrAlYN coating had better compactness, a flat and smooth surface, and a smaller friction coefficient, so it reduced friction and significantly reduced drilling force. The drilling force increased suddenly in the curve due to the breakage of the drilling edge, resulting in a discontinuous drilling edge and large roughness.

(2) Drilling temperature comparison

In the process of drilling, the maximum temperature of the drilling area was recorded through an infrared thermal imager, and then the maximum temperature under different working conditions was compared and analyzed. The temperature of the main cutting edge when the drill bit and the workpiece interacted violently could not be measured by the thermal imager, but the cutting heat was usually taken out by the chips. Too-high heat could even make the bit turn red. Therefore, the comparison of the trend of the drilling temperature detected under the same experimental conditions was quite significant.

**Figure 7:**
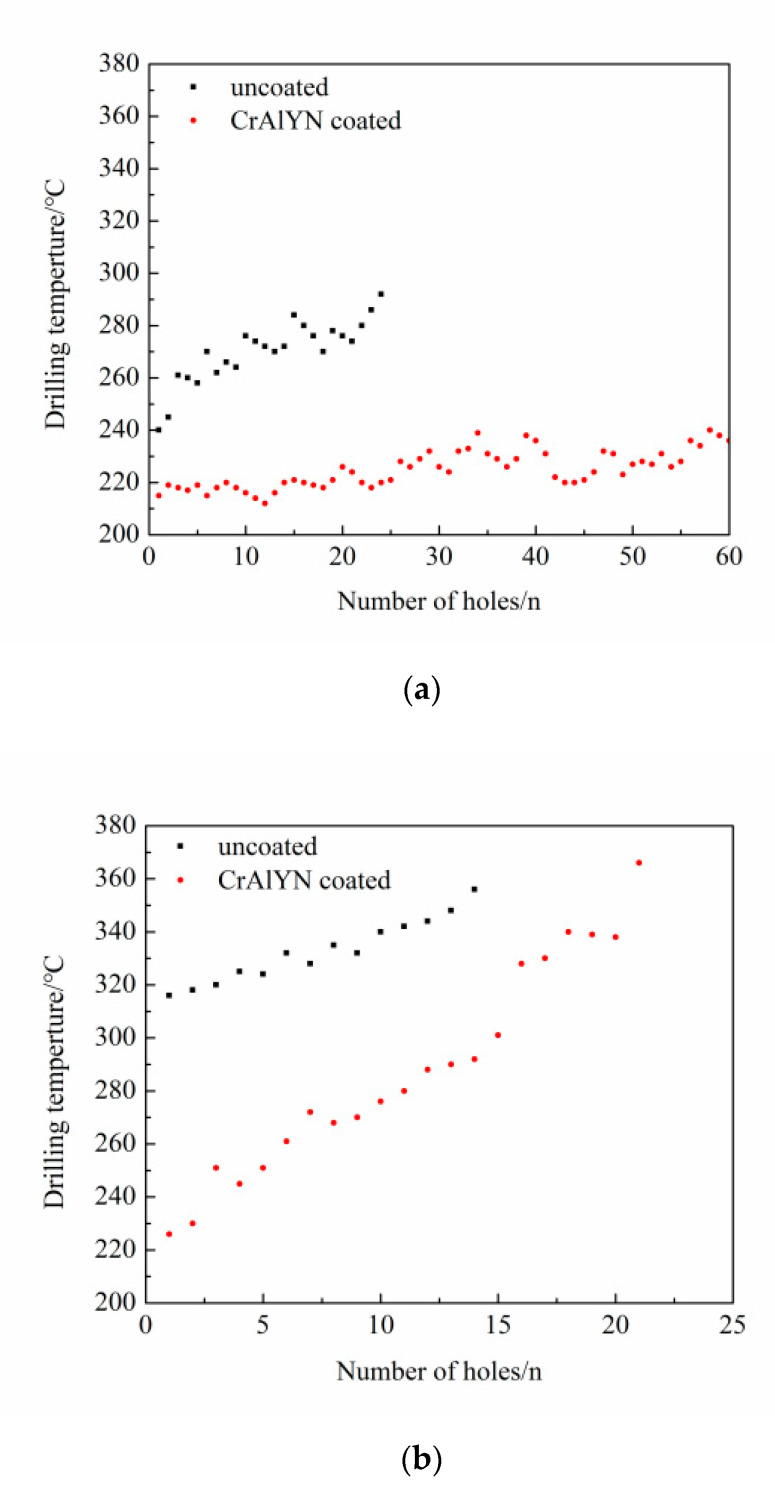
Comparison of drilling temperatures between uncoated and coated bits (drilling depth of 6 mm and drilling feed rate of 0.04 mm/r). (**a**) Drilling temperatures at a drilling speed of 15 m/min. (**b**) Drilling temperatures at a drilling speed of 20 m/min.

Figure 7 shows the comparison of the drilling temperature of CrAlYN coated and uncoated YG10 bit in the two working conditions. Obviously, at lower rotational speeds, the drilling temperature of the coated bit was stable and more than 15% lower than that of the uncoated bit. At higher rotational speeds, the drilling temperature of the coated bit was more than 20% lower than that of the uncoated bit in the initial drilling phase, but the temperature increase was significantly greater than that of the uncoated bit in the later drilling phase, and the drilling temperature was similar to that of the uncoated bit when the bit failed. On the one hand, CrAlYN coating had a smaller friction coefficient, so the coated bit friction, drilling force, and heat generation. On the other hand, CrAlYN coating had low thermal conductivity, so less heat was transferred to the tool body, making the temperature rise slowly and delaying the tool damage, thus prolonging the service life. The higher the drilling speed was, the more heat was generated; the insulation effect of the coating reduced the heat entering the tool. In addition, the nickel-based superalloy had poor thermal conductivity, resulting in more concentrated heat, faster temperature increase, and serious bonding.

### 3.3. Bit Wear Morphology

**Figure 8:**
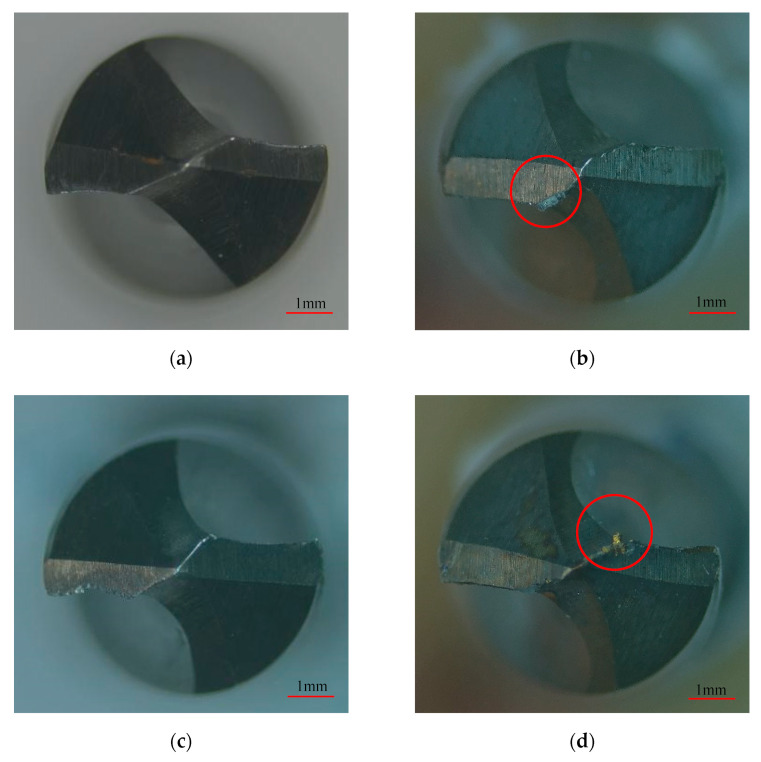
Bit failure wear morphology. (**a**) Uncoated bit at drilling speed of 15 m/min. (**b**) Coated bit at drilling speed of 15 m/min. (**c**) Uncoated bit at drilling speed of 20 m/min. (**d**) Coated bit at drilling speed of 20 m/min.

Figure 8 shows the morphology of the main rear tool surface of the bit observed under the tool microscope. The main cutting edge of the uncoated bit is worn and broken seriously in Figure 8c. Compared with the uncoated bit, bonding was clearly displayed at the coated bit, and the sticking substance looked yellow at higher drilling speed (the red circle in Figure 8d), indicating that the drilling temperature increased significantly with the increase in drilling speed.

**Figure 9:**
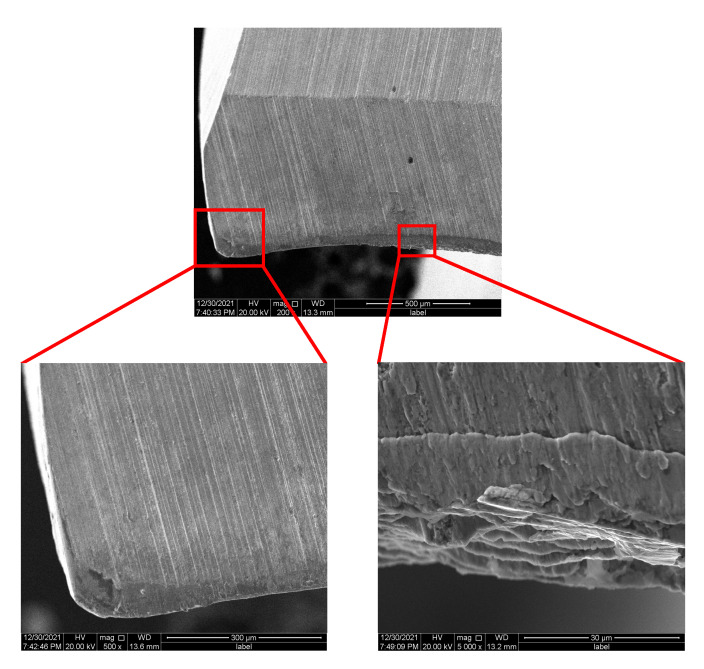
Crack in back of uncoated drill.

**Figure 10:**
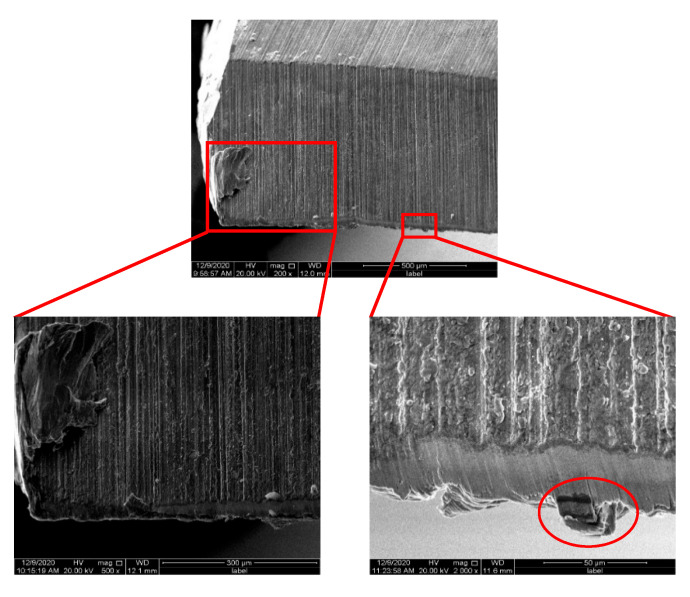
Wear morphology of rear edge of main cutting edge of coated drill.

Figure 9 and Figure 10 show the wear morphology of the main rear tool surface of the uncoated bit and CrAlYN coated bit photoed by SCANNING electron microscope (SEM). As shown in Figure 9, the contact between the main drilling edge and the auxiliary drilling edge of the bit was severely worn or even broken because the outer edge of the main drilling edge had the highest linear velocity and the largest extrusion pressure. 

As shown in Figure 10, CrAlYN coated bits had longer service life, but the wear zone behind the tool surface was smaller than that of uncoated bits, and the edge of the wear zone was continuous and neat. Although the wear zone was removed due to severe wear during the drilling, the coating in other areas still protected the bit base. Thus, CrAlYN coating had good bonding with the bit matrix, and strong oxidation resistance could slow down the diffusion of oxygen atoms in the coating caused by temperature rise in the process of friction and wear. It slowed down the oxidation of the coating and matrix materials, prevented premature oxidation cracking, realized oxidation inhibition, wear resistance, and heat insulation, and thus effectively improved the cutting performance of CrAlYN coated bits and prolonged tool life.

A serious bonding phenomenon was seen at the main rear tool surface of the uncoated bit in Figure 9. In the metal drilling process, continuous chips flowed through the main cutting edge and had friction on the contact surface. At the same time, under the action of drilling heat, the hardness of workpiece material at the contact area decreased, resulting in plastic deformation and element diffusion of drill and chip. In the end, the tool and chip were closely bound, causing “retention”. At the end of one drilling, the temperature dropped, the chips were bonded to the drilling edge, and plastic deformation occurred again when drilling again. Therefore, the cracks on the main rear edge of the uncoated bit were bonded, layered by layered, and irregular. By comparison, the main drilling edge of the CrAlYN coating bit was more slightly bonded, but there were also lumps (see the red circle in Figure 10).

**Figure 11:**
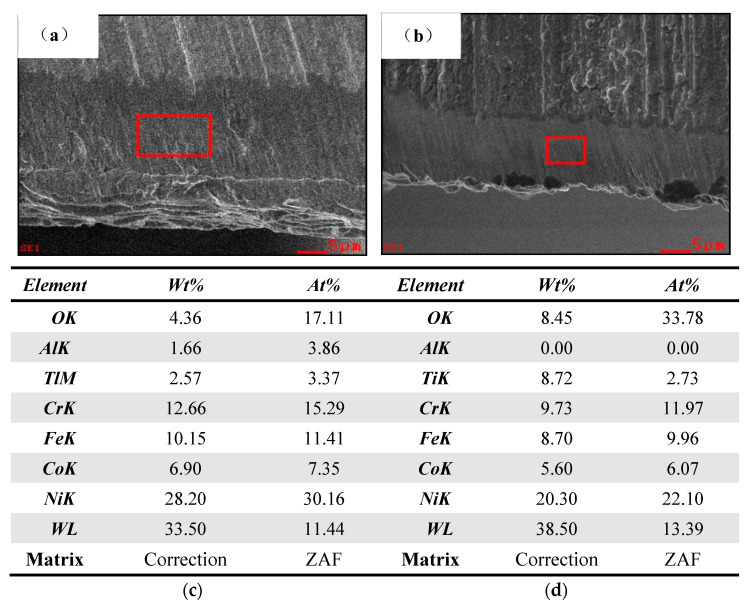
Energy spectrum analysis of the wear zone of drill rear drill surface. (**a**) Main rear tool surface of the uncoated bit. (**b**) Main rear tool surface of the CrAlYN coated bit (**c**) Element composition list of uncoated bit rear tool face. (**d**) Element composition list of coated bit rear tool face.

Figure 11 shows the energy spectrum analysis of the wear zone of the main rear tool surface of the uncoated bit and the CrAlYN coated bit, respectively. The Ni, Fe, and Cr elements were obviously high, indicating that the workpiece material was bonded on the main rear tool surface. Combined with the analysis in Figure 9, it can be seen that bonding wear is the main failure mode of cemented carbide bit drilling nickel-based superalloy. Compared with uncoated bits, the wear zone coating of CrAlYN coated bits was damaged, and the carbide matrix was exposed, but the wear zone was obviously smaller than that of uncoated bits. When the tool life of CrAlYN coated bit is much longer than that of uncoated bit, the Ni, Fe, and Cr elements in the wear zone of CrAlYN coated bits were slightly lower than that of uncoated bits. CrAlYN coated bits could retard tool wear and prolong tool service life.

**Figure 12:**
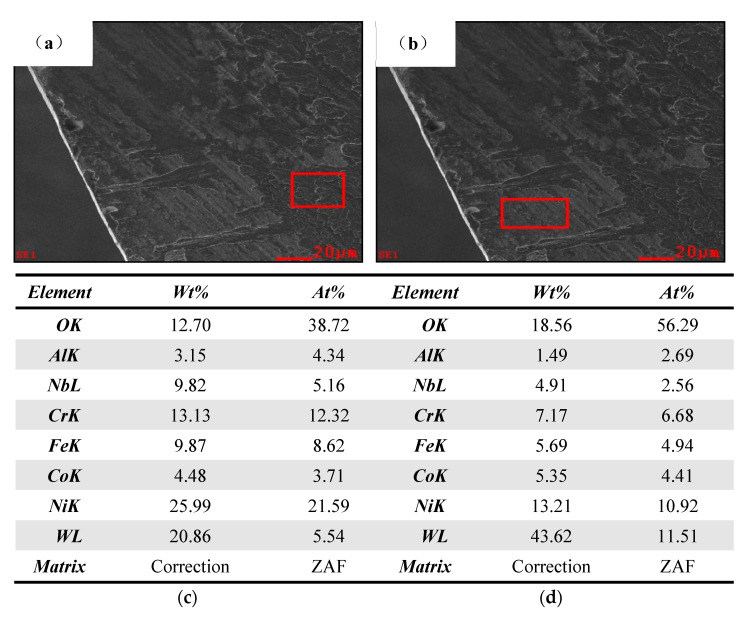
Adhesive wear EDS on the front edge of coated drill. (**a**) Bonded area on the front surface of the CrAlYN coated bit. (**b**) Coating falling off area on the front surface of the CrAlYN coated bit. (**c**) Element composition list in the figure. (**d**) Element composition list in the figure.

Figure 12 shows the wear morphology of CrAlYN coating on the front surface of the bit. As seen from it, Ni, Fe, and Cr elements in the red box in Figure 12a have a significantly high proportion. The plastic deformation layer at the face of the chip slowed down. When the slowness and pressure increased to a certain extent, the underlying chip and tool front surface bonded, called the bonded area of coated bit front surface. Moreover, the Co and W contents in the red box in Figure 12b were high because the chip flow and extrusion took away the bond, and the coating fell off, called the coating falling off area on the front tool of the coated bit.

It can be seen that the failure of CrAlYN coated carbide bits was mainly caused by the bonding wear of the rear and front tool surfaces. The coating protected the carbide bit base and improved the bit service life, but once the coating fell off, the bonding increased, and its failure was similar to that of uncoated carbide bits.

### 3.4. The Machined Surface Quality

**Figure 13:**
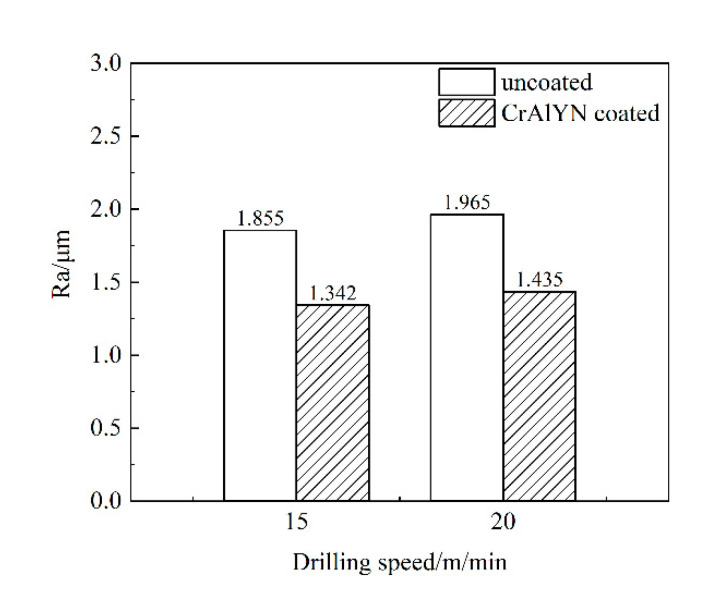
Inner surface roughness Ra.

The inner surface roughness Ra of all holes was measured by the TR200 surface roughness meter, and the average value was calculated, as shown in Figure 13. Under different drilling speeds, the inner surface roughness of the coated bit was more than 25% lower than that of the uncoated carbide bit because CrAlYN coated bits had a flat, smooth surface that had less friction and had slightly higher machining quality than uncoated bits. At low-speed working conditions, the machined surface roughness was slightly lower. Because of lower drilling temperature, longer tool service life, and the processing quality was slightly higher than that at the high-speed working condition.

## 4. Conclusions

In this paper, the orthogonal test was conducted on the carbide bit YG10 dry drilling nickel-based superalloy to analyze the primary and secondary factors influencing the bit service life, drilling forces, and drilling temperature under dry drilling conditions. Secondly, under the selected working conditions, the self-made CrAlYN coated carbide bit and uncoated carbide bit were compared and analyzed from perspectives of bit service life, drilling forces, drilling temperature, bit surface topography, failure mechanism, and machined surface quality. The wear morphology and machined surface quality of the CrAlYN coated carbide bit and uncoated carbide bit were compared. Finally, the following conclusions were reached:

(1) In the dry drilling condition, the primary factor influencing the dry drilling of nickel-based superalloy of carbide bit is the drilling depth, which is mainly reflected by the drilling temperature. At the drilling speed of 15 m/min, the bit has a longer service life, lower drilling temperature, smaller drilling force, and more stable drilling state;
(2) At lower rotation speeds, the CrAlYN coating can protect the carbide bit matrix and significantly prolongs the bit service life. To be specific, it can prolong the bit service life by 250% at a drilling speed of 15 m/min and 50% at a drilling speed of 20 m/min;
(3) CrAlYN coating can reduce drilling forces and drilling temperature, retard the formation and propagation of surface cracks, delay tool wear, and improve the quality of a machined surface;
(4) The failure mode of coated cemented carbide bit is similar to that of the uncoated cemented carbide bit after the CrAlYN coating falls off in the wear zone of the carbide bit, which is mainly bonding wear on the rear tool surface and the front tool surface.

## Figures and Tables

**Table 1:** Chemical composition of nickel-base superalloy GH4169.

Element	Ni	Cr	Nb	Mo	Ti	C	Si	Mn	B	Fe
Mass/%	51.75	17	5.15	2.93	1.07	0.042	0.21	0.03	0.006	Maargin

**Table 2:** Material properties of workpiece.

Elastic Modulus (GPa)	Poisson’s Ratio μ	Density ρ(Kg/m^3^)	Johnson–Cook Model Parameters						
			A(MPa)	B(MPa)	C	n	m	Tmelt(℃)	Troom(℃)
220	0.3	8420	985	949	0.01	0.4	1.65	1320	20

**Table 3:** Mass fraction of each element in the drill.

Cutting Tool Serial No.	Element Mass Fraction (%)		
	W	Co	C
YG10	84	10	6

**Table 4:** Mechanical properties of bit materials.

Cutting Tool Serial No.	Mechanical Properties					
	Density g/cm^−3^	Hardness HRA	Bending Strength MPa	Compressive Strength MPa	Elastic Modulus GPa	Impact Toughness J/cm^−2^
YG10	14.7	88.5	2700	4700	585	2.8

**Table 5:** Element content of CrAlYN coating.

Coating	Element Content (%)			
	Cr	Al	Y	N
CrAlYN	44.71	9.72	3.28	42.29

**Table 6:** Factor level.

Level	Factor		
	Drilling Speed (m/min)	Drilling Feed Rate (r/mm)	Drilling Depth (mm)
**1**	15	0.03	3
**2**	20	0.04	6
**3**	25	0.05	9

**Table 7:** Orthogonal drilling experiment results.

Serial No.	Drilling Speed	Feed Rate (mm/r)	Drilling Depth (mm)	Number of Drilling	Drilling Force (N)	Drilling Tem.
	(m/min)			(n)		(°C)
**1**	15	0.03	3	36	486	260
**2**	15	0.04	6	24	1192	291
**3**	15	0.05	9	12	863	291
**4**	20	0.03	6	18	578	248
**5**	20	0.04	9	6	1426	369
**6**	20	0.05	3	16	1022	302
**7**	25	0.03	9	4	1736	363
**8**	25	0.04	3	60	885	311
9	25	0.05	6	6	1821	378

**Table 8:** Range analysis of bit service life.

Level	Drilling Speed			Feed Rate			Drilling Depth		
	15 m/min	20 m/min	25 m/min	0.03 mm/r	0.04 mm/r	0.05 mm/r	3 mm	6 mm	9 mm
**Ki**	72	40	70	58	90	34	112	48	22
**ki**	24	13	23	19	30	11	37	16	7
**Range**	11			19			30		
**Order: Drilling depth > Feed rate > Drilling speed**									
**Optimization scheme: drilling speed: 15 m/min; feed rate: 0.04 mm/r; drilling depth: 3 mm**									

**Table 9:** Range analysis of drilling force.

Level	Drilling Speed			Feed Rate			Drilling Depth		
	15 m/min	20 m/min	25 m/min	0.03 mm/r	0.04 mm/r	0.05 mm/r	3 mm	6 mm	9 mm
**Ki**	2541	3026	4442	2800	3503	3706	2393	3591	4025
**ki**	847	1008.67	1480.67	933.33	1167.67	1235.33	797.67	1197	1341.67
**Range**	633.67			302			544		
**Order: Drilling speed > Drilling depth > Feed rate**									
**Optimization scheme: drilling speed: 15 m/min; feed rate: 0.03 mm/r; drilling depth: 3 mm**									

**Table 10:** Range analysis of drilling temperature.

Level	Drilling Speed			Feed Rate			Drilling Depth		
	15 m/min	20 m/min	25 m/min	0.03 mm/r	0.04 mm/r	0.05 mm/r	3 mm	6 mm	9 mm
**Ki**	842	919	1052	871	971	971	873	917	1023
**ki**	280.67	306.33	350.67	290.33	323.67	323.67	291	305.67	341
**Range**	70			33.34			50		
**Order: Drilling depth > Feed rate> Drilling speed**									
**Optimization scheme: drilling speed: 15 m/min; feed rate: 0.04 mm/r; drilling depth: 3 mm**									

## Data Availability

The data used to support the findings of this study are included within the article.
